# Effects and acceptability of implementing improved cookstoves and heaters to reduce household air pollution: a FRESH AIR study

**DOI:** 10.1038/s41533-019-0144-8

**Published:** 2019-08-15

**Authors:** Frederik van Gemert, Corina de Jong, Bruce Kirenga, Patrick Musinguzi, Shamim Buteme, Talant Sooronbaev, Aizhamal Tabyshova, Berik Emilov, Maamed Mademilov, Pham Le An, Nguyen Nhat Quynh, Tran Ngoc Dang, Le Huynh Thi Cam Hong, Ryan Chartier, Evelyn A. Brakema, Job F. M. van Boven, FRESH AIR, Janwillem Kocks, Rebecca Nantanda, Winceslaus Katagira, Grace Ndeezi, James Tumwine, Simon Walusimbi, Azamat Akylbekov, Pham Duong Uyen Binh, Tran Diep Tuan, Le Thi Tuyet Lan, Tran Thanh Duv Linh, Kim Xuan Loan, Le Thanh Van, Nguyen Nhu Vinh, Niels H. Chavannes, Rianne M. J. J. van der Kleij, Charlotte Poot, Marilena Anastasaki, Antonios Bertsias, Vasiliki E. Chatzea, Christos Lionis, Sophia Papadakis, Dimitra Sifaki-Pistolla, Ioanna Tsiligianni, Sally Singh, Dennis Burges, Ben Hedrick, James Stout, Louise Warren, Irene Ferarrio, Pippa Powell, Andy Barton, Lucy Cartwright, Sanne van Kampen, Rupert Jones, Jillian Pooler, Anja Poulsen, Jesper Kjærgaard, Nick Hopkinson, Liza Cragg, Hilary Pinnock, Sian Williams, Andy McEwen, Susanne Reventlow, Marianne Stubbe-Østergaard, Maarten J. Postma, Jaime Correia de Sousa

**Affiliations:** 1 University of Groningen, University Medical Center Groningen, Groningen Research Institute for Asthma and COPD (GRIAC), Department of General Practice & Elderly Care Medicine, Groningen, The Netherlands; 2 University of Groningen, University Medical Center Groningen, Department of Health Sciences, Unit of Global Health, Groningen, The Netherlands; 3 Department of Medicine and Makerere University Lung Institute, Makerere University, Kampala, Uganda; 4 Pulmonary Department, National Center of Cardiology and Internal Medicine, Bishkek, Kyrgyzstan; 5 Center of Training Family Medicine, University of Medicine and Pharmacy at Ho Chi Minh City, Ho Chi Minh City, Vietnam; 6 Department of Environmental Health, University of Medicine and Pharmacy at Ho Chi Minh City, Ho Chi Minh City, Vietnam; 7 RTI International, Research Triangle Park, NC USA; 8 Department of Public Health and Primary Care, Leiden University Medical Center, Leiden, the Netherlands; 9 Clinic of Social and Family Medicine, School of Medicine, University of Crete, Heraklion, Greece; 10 International Primary Care Respiratory Group, London, UK; 11 Coventry University, Coventry, UK; 12 Department of Pediatrics, University of Washington School of Medicine, Seattle, WA USA; 13 European Lung Foundation, Sheffield, UK; 14 Faculty of Medicine and Dentistry, University of Plymouth, Plymouth, UK; 15 Global Health Unit, The Department of Paediatrics and Adolescent Health, Juliane Marie Center, Copenhagen University Hospital “Rigshospitalet”, Copenhagen, Denmark; 16 Research Unit for General Practice and Section of General Practice, Department of Public Health, Copenhagen University, Copenhagen, Denmark; 17 Imperial College London, London, UK; 18 University of Edinburgh, Usher Institute of Population Health Sciences and Informatics, Edinburgh, UK; 19 National Centre for Smoking Cessation and Training, Dorchester, UK; 20 University of Groningen, University Medical Center Groningen, Groningen, the Netherlands; 21 University of Minho, School of Medicine, Braga, Portugal

**Keywords:** Public health, Epidemiology

## Abstract

The objective was to evaluate the effectiveness and acceptability of locally tailored implementation of improved cookstoves/heaters in low- and middle-income countries. This interventional implementation study among 649 adults and children living in rural communities in Uganda, Vietnam and Kyrgyzstan, was performed after situational analyses and awareness programmes. Outcomes included household air pollution (PM_2.5_ and CO), self-reported respiratory symptoms (with CCQ and MRC-breathlessness scale), chest infections, school absence and intervention acceptability. Measurements were conducted at baseline, 2 and 6–12 months after implementing improved cookstoves/heaters. Mean PM_2.5_ values decrease by 31% (to 95.1 µg/m^3^) in Uganda (95%CI 71.5–126.6), by 32% (to 31.1 µg/m^3^) in Vietnam (95%CI 24.5–39.5) and by 65% (to 32.4 µg/m^3^) in Kyrgyzstan (95%CI 25.7–40.8), but all remain above the WHO guidelines. CO-levels remain below the WHO guidelines. After intervention, symptoms and infections diminish significantly in Uganda and Kyrgyzstan, and to a smaller extent in Vietnam. Quantitative assessment indicates high acceptance of the new cookstoves/heaters. In conclusion, locally tailored implementation of improved cookstoves/heaters is acceptable and has considerable effects on respiratory symptoms and indoor pollution, yet mean PM_2.5_ levels remain above WHO recommendations.

## Introduction

Worldwide, almost three billion people, mostly from low- and middle-income countries (LMICs), rely on open fires and burning of biomass fuels (wood, animal dung, crop residues, straw and charcoal) for cooking and heating.^1,2^ Notably, people living in poverty are unable to afford clean fuels and efficient cooking practices, and have the greatest exposure to household air pollution (HAP).^3,4^

Exposure to HAP for cooking and heating causes almost four million premature deaths each year, mostly in LMICs,^5–7^ http://www.who.int/news-room/fact-sheets/detail/household-air-pollution-and-health. Besides being linked to chronic obstructive pulmonary disease (COPD), exposure to HAP is associated with a wide range of other health-damaging outcomes.^1,6,7^ There is evidence that biomass smoke increases the risk of pneumonia in children, eye disease (e.g. cataract), low birthweight and lung cancer, and affects cardiovascular, metabolic and cognitive health throughout the life course.^1,2,7,8^

To combat the burden of HAP-related respiratory and non-respiratory diseases in LMICs, preventive actions are urgently needed. Reducing HAP encompasses three types of interventions: at the source of smoke, directed towards the living environment, and aimed at the user.^9^ Over the past three decades, multiple intervention programmes to reduce exposure to HAP have been conducted. Initially, the drive behind these programmes was preventing deforestation and encouraging local economic development, rather than reducing health risks from HAP.^6,10^ Some national programmes did make a transition to cleaner fuels including liquefied petroleum gas (LPG) as their socioeconomic circumstances improved.^11^ Yet, the poorest people in rural areas still have limited opportunities to switch to clean fuels, and remain dependent on improved cookstoves and/or heaters as the sole technological option for reducing HAP exposure.^11,12^

So far, studies towards the effect of improved cookstoves on health and pollution reported variable findings.^6,10–14^ Findings on health outcomes were not convincing, although symptom relief was often reported.^12,14^ Reduction of HAP was often achieved, but the pollutant levels remained well above the limits as reported by the WHO air quality guideline.^10,11,15^ One of the factors explaining the variable findings was the difficulty with acceptability, implementation and sustained use of the improved stoves by its users. ^10,13,16^ Of note, rural communities were often not aware of the detrimental effects of HAP exposure.^4,17^ We hypothesise that implementation of improved cookstoves and heaters, embedded in HAP awareness programmes and tailored to local context and needs, would result in higher acceptance, and enhanced and maintained respiratory health effects.

The aim of this study was to evaluate the acceptability and effectiveness of a locally tailored implementation strategy for the introduction of improved cookstoves and heaters in selected communities of three LMICs: Uganda, Vietnam and Kyrgyzstan.

## Results

### Study population characteristics

Initially, a total of 649 participants were recruited in this study. This number comprised 360 adults and 289 children (Tables 1 and 2). Mean adult age ranged from 36.2 years (SD 11.6) in Uganda to 52.1 years (SD 15.6) in Vietnam, while for children this ranged from 2.8 years (SD 1.6) in Vietnam to 8.7 years (SD 4.4) in Kyrgyzstan. One third to half of the participants were male. Eventually, a total of 610 participants (335 adults and 275 children) completed the study. The main reason for drop-out (~80%) was that the family members moved to another village or district (often caused by divorce); sometimes participants were not at home or not available due to illness or childbirth.

**Table 1:** Health outcomes of adults in Uganda, Vietnam and Kyrgyzstan

Adults	Uganda			Vietnam			Kyrgyzstan		
	Baseline	1st post	2nd post	Baseline	1st post	2nd post	Baseline	1st post	2nd post
N (absolute)	185		168	137		129	38		38
Age (years)	36.2	37.2	37.6	52.1	52.5	52.8	42.1	42.3	43.1
Gender male	47.3	47.1	46.1	34.6	33.1	27.1	36.8	47.1	44.7
Daily respiratory symptoms									
Cough	33.0	7.0	0	59.9	50.9	40.3	68.4	17.6	13.2
Phlegm	30.8	5.2	0	39.4	27.7	26.4	47.4	14.7	18.4
Wheeze	10.8	2.9	0	33.8	28.5	29.5^†^	26.3	5.9	2.6
Breathlessness	18.4	2.4	0	39.0	30.0	35.7^†^	36.8	0	0
CCQ (total score)	0.38	0.13	0.04	1.26	0.98	1.02	1.22	0.38	0.09
Symptoms during cooking									
Cough	51.6	16.3	5.4	41.6	23.0	18.8	73.7	5.9	0
Wheeze	10.1	4.1	0	16.1	6.3	8.6†	31.6	0	0
Headache	42.1	15.1	1.8	32.8	10.2	12.5	71.1	8.8	0
Irritated eyes	65.0	20.3	31.5	56.2	21.1	25.8	76.3	0	5.3
Watery eyes	76.8	30.3	32.7	72.3	75.8	64.8^†^	63.2	0	0
Nasal congestion	37.6	10.5	11.3	44.5	18.3	19.5	60.5	0	0
Running nose	65.0	19.3	26.2	47.4	28.9	31.3	52.6	0	0
Irritated throat	35.4	2.9	1.2	16.8	3.1	6.3	63.2	2.9	0
Fatigue	28.9	6.4	0	32.1	4.7	11.7	68.4	0	0
MRC-breathlessness score	1.16	1.08	1.02	1.61	1.78	1.54^†^	1.71	1.15	1.32
Chest infections^a^									
None	72.4	90.6	94.0	93.4	99.2	92.2^†^	36.8	35.3	92.1
1	13.3	7.0	5.4	2.2	0.8	3.9	47.4	64.7	7.9
2 or more	14.1	2.3	0.6	4.4	0	3.9	15.8	0	0

All data are percentages unless stated otherwise; *p-*values between baseline and 2nd post are all statistically significant (*p* < 0.05), except for data marked with dagger, which are ‘not significantʼ

*CCQ* Clinical COPD Questionnaire, *MRC* Medical Research Council

^a^Chest infections during last 6 months

**Table 2:** Health outcomes of children in Uganda, Vietnam and Kyrgyzstan

Children	Uganda			Vietnam			Kyrgyzstan		
	Baseline	1st post	2nd post	Baseline	1st post	2nd post	Baseline	1st post	2nd post
N (absolute)	141		128	87		86	61		61
Age (years)	3.0	3.8	4.1	2.8	31	3.6	8.7	9.4	9.7
Gender male	51.1	50.0	50	45.3	44.6	42.6	50.8	50.9	50.9
Daily respiratory symptoms									
Cough	30.9	12.7	0	77.0	55.3	45.6	36.8	0	1.9
Phlegm	18.7	6.0	0	41.9	24.7	17.6	36.4	0	1.9
Wheeze	3.6	1.5	0	51.7	31.8	25.0	9.1	0	0
Dyspnoea	7.9	1.5	0	33.3	10.7	11.9	20.5	0	0
Symptoms during cooking									
Cough	46.0	12.7	8.1	35.6	17.4	15.9	46.7	1.8	0
Wheeze	3.6	2.2	0	23.0	9.3	8.7	5.0	0	0
Headache	12.9	3.7	0	12.6	0	7.2^†^	36.7	1.8	0
Irritated eyes	48.2	15.7	25.4	35.6	5.8	18.8	46.7	0	0
Watery eyes	72.7	22.4	28.5	47.1	36.5	39.7^†^	72.7	0	0
Nasal congestion	24.5	6.7	9.8	26.4	10.5	8.7	31.7	0	0
Running nose	64.7	14.2	26.8	36.8	15.1	17.4	40.0	0	0
Irritated throat	35.4	16.5	3.0	6.7	2.3	2.9^†^	30.0	0	0
Fatigue	0.7	0	0	11.5	2.3	1.4	16.7	0	0
Missed days at school^a^	3.0	3.7	1.0	0.8	1.1	1.9	3.43	0.47	0.95
Chest infections^b^									
None	80.6	89.6	89.4	80.5	95.3	85.1^†^	15.9	27.3	83.0
1	10.8	6.7	6.5	8.0	1.2	4.7	54.5	69.1	13.2
2 or more	8.6	3.7	4.1	11.5	3.5	10.2	29.5	3.6	3.8

All data are percentages unless stated otherwise; *p-*values between baseline and 2nd post are all statistically significant (*p* < 0.05), except for data marked with dagger, which are ‘not significantʼ

^a^School days missed during last 3 months

^b^Chest infections during last 6 months

### Situational analysis

Each setting had a different traditional way of cooking, generally using different traditional cookstoves/heaters and different biomass fuels. In Uganda and Vietnam, everybody used solid fuels for cooking; under specific circumstances, such as cold nights, the cookstoves were used as heaters as well (mentioned by 64% of households in Uganda and 10% in Vietnam). In Kyrgyzstan, 95% of households used solid fuels for cooking and 50% for heating (Supplementary Table 1). Of note, in Vietnam, tobacco smoking inside houses was common in 68% of the households. In contrast, in Uganda, this was 24% and in Kyrgyzstan 20%. Specific cooking and heating habits are provided below.

#### Uganda

A total of 88% reported using an open fire with wood as main solid fuel, but grass, twigs, crop residues and charcoal were used to light the fire. Women reported spending 4 h a day cooking indoors and 2.5 h outdoors. Chimneys were not used at all, but in 8.1% of households, a hood was used to help ventilate the smoke.

#### Vietnam

A total of 70% used a surrounded fire (fire is partially or completely surrounded by mud, clay or other materials) with wood as main solid fuel. Almost 72% of households used LPG as a secondary stove, often to light grass, twigs, and crop residues to get their primary stove started or to reheat the food. The women cooked 1.9 h a day indoors and 0.5 h outdoors. Chimneys were used in 17.5% of households and a hood in 1.3%.

#### Kyrgyzstan

A total of 95% used an improved multiple pot stove and 5% a single pot stove. All participants used either wood, dung or coal as their primary fuel. Coal was primarily used during the winters and (yak) dung more frequently in the poorest households. During the short summer, 15% of households used an electric stove as a secondary stove. Women spent 1.7 h a day cooking indoors and 0.6 h outdoors. All the cookstoves were used as heaters, meaning the stoves were used the whole day through during the cold season. All participating households used a built-in chimney to vent the smoke from cooking/heating out of the home.

### Environmental outcomes

**Fig. 1:**
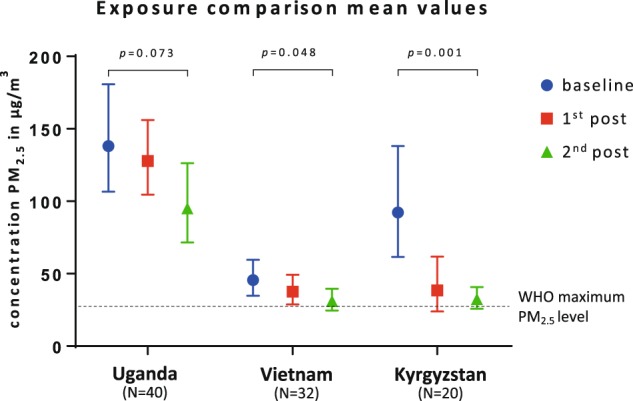
Mean PM_2.5_ measurements in Uganda, Vietnam and Kyrgyzstan before and after cookstove/heater intervention. The mean PM_2.5_ with 95% confidence interval; baseline measured before intervention, first post 2 months and second post 6 months (in Uganda and Vietnam) or 12 months (in Kyrgyzstan); *p*-value is measured between baseline and second post

Environmental outcomes (i.e. pollutants) are presented in Fig. 1 and Table 3. At baseline, the geometric mean PM_2.5_ levels in all countries were high, all above the WHO air quality guidelines (25 µg/m^3^ for 24 h mean).^15^ More specifically: in Uganda levels were 138.1 µg/m^3^ (95%CI: 106.6–180.8), in Vietnam 45.6 µg/m^3^ (95%CI: 34.8–59.6) and in Kyrgyzstan 92.3 µg/m^3^ (95%CI: 61.6–138.1) (Fig. 1).

**Table 3:** PM_2.5_ of 99th, 95th and 30th percentile and CO-measurements from Uganda, Vietnam and Kyrgyzstan

	Uganda			Vietnam			Kyrgyzstan		
	Baseline	1st post	2nd post	Baseline	1st post	2nd post	Baseline	1st post	2nd post
PM_2.5_ 99th percentile									
** Mean**	**2421.9**	**1778.6**	**2157.1**	**912.6**	**529.2**	**475.8**	**1634.3**	**559.6**	**255.1**
Max	10493.0	12096.8	15477.4	10176.3	4985.0	5017.2	6695.4	3038.5	1484.5
Min	194.6	164.3	41.6	43.1	14.6	34.7	98.9	51.1	30.9
PM_2.5_ 95th percentile									
** Mean**	**434.7**	**388.9**	**243.6**	**116.1**	**128.2**	**80.9**	**509.9**	**260.2**	**106.4**
Max	2565.2	2064.4	2694.5	425.7	1229.0	314.4	2593.5	1524.8	467.9
Min	83.0	93.0	31.3	24.0	14.3	13.7	60.9	35.0	26.1
PM_2.5_ 30th percentile									
** Mean**	**35.4**	**48.7**	**41.7**	**15.2**	**18.1**	**10.9**	**23.4**	**7.8**	**16.2**
Max	168.4	295.1	162.1	73.5	62.0	29.7	82.2	14.5	30.6
Min	5.1	9.2	0.7	1.0	4.2	2.6	8.1	4.1	4.5
CO									
** Mean**	**2.51**	**1.83**	**1.42**	**0.68**	**0.55**	**0.42**	**3.86**	**1.16**	**1.77**
Max	133.5	86.5	91.9	112.9	44.1	103.8	94.9	42.5	12.7
CO adults									
** Mean**	**2.75**	**2.12**	**1.30**	**1.11**	**0.56**	**0.34**	**3.37**	**1.02**	**1.40**
CO children									
** Mean**	**2.08**	**1.36**	**1.74**	**0.24**	**0.54**	**0.50**	**4.62**	**1.34**	**1.92**

PM_2.5_ (particulate matter smaller than 2.5 µg in µg/m^3^ and CO in ppm (part per million); mean values are in bold; the max (maximum) and min (minimum) are related to the mean value

*Post* post-intervention

After the intervention, mean PM_2.5_ exposures decreased in all countries as shown in Fig. 1: in Uganda to 95.1 µg/m^3^ (95%CI: 71.5–126.6), a total decrease of 31% (*p* = 0.073), in Vietnam to 31.1 µg/m^3^ (95%CI: 24.5–39.5), a total decrease of 32% (*p* = 0.048) and in Kyrgyzstan to 32.4 µg/m^3^ (95%CI: 25.7–40.8), a total decrease of 65% (*p* = 0.001).

The 99th (the highest 30 min of exposure) and 95th (the highest 2.5 h of exposure) percentile values display the concentration PM_2.5_ with the maximum and minimum range (Table 3). The 30th percentile values display the background mean PM_2.5_ (~33.5 h of the 48 h measurement were above this value). After the intervention, all the mean PM_2.5_ exposures remained above the WHO air quality guidelines. The variation between the countries was high.

At baseline, the mean CO-levels were all below the WHO air pollution guidelines (30 mg/m^3^ or 26.6 ppm for 1 hour mean and 7 mg/m^3^ or 6.1 ppm for 24 h mean).^15^ The mean CO values decreased in all countries. All mean CO values remained below the WHO air quality guidelines. Of note, this was not the case for the maximum values, except for Kyrgyzstan.

### Clinical outcomes

In all three countries at baseline, both adults and children reported high rates of *daily respiratory symptoms* and *symptoms during cooking*, with the highest values occurring in Vietnam and Kyrgyzstan (Tables 1 and 2). The number of (self-reported) chest infections was particularly high in Kyrgyzstan.

Following the introduction of the improved cookstoves/heaters, these were the health outcomes (Tables 1 and 2):

#### Uganda

All reported *daily respiratory symptoms* eventually disappeared completely among adults and children. *Symptoms during cooking* decreased and several complaints (wheeze, fatigue) disappeared completely. *CCQ* and *MRC* score decreased by 89 and 12%, respectively. The number of *missed days at school* among children decreased by 67% between the baseline and second postintervention measurement. All changes in Uganda between the baseline and second postintervention measurement were statistically significant (*p* < 0.05).

#### Vietnam

There was a decrease in *daily respiratory symptoms* among adults and children, but the reduction of several symptoms among adults was not significant. The same pattern was seen with *symptoms during cooking* for both adults and children: there was a slight decrease of several symptoms, but other symptoms were not significantly reduced. The total *CCQ score* reduced by 32%, but the *MRC score* did not change significantly. The number of *missed days at school* among children even increased.

#### Kyrgyzstan

The *daily respiratory symptoms* among adults and children decreased; breathlessness among adults and both breathlessness and wheeze among children disappeared. *Symptoms during cooking* disappeared in almost all cases, both in adults and children. The *CCQ* and *MRC score* reduced by 93 and 23% respectively. The number of *chest infections* decreased considerably for both adults and children. The number of *missed days at school* decreased by 72%. All changes in Kyrgyzstan between the baseline and second postintervention measurement were statistically significant (*p* < 0.05).

### Implementation outcomes and process evaluation

The dimensions of the RE-AIM framework guide the evaluation of the several aspects of the intervention.^18–20^ In *Reach*, the households with the improved cookstoves (and assessed with the questionnaires) were randomly chosen, and within this group, four households were randomly chosen to have their personal exposure to PM_2.5_ and CO measured. All residents of the selected households, without exception, agreed to join the study. *Adoption* describes the choice of the different cookstoves/heaters made by the energy providers working for years in the concerned country in close collaboration with the local FRESH AIR team (Supplementary Fig. 1). The energy providers gave simple yet detailed demonstrations to the community how the improved cookstoves/heaters worked. The people in Uganda and Vietnam had to pay for the improved cookstove but received a compensation (approximately the price of the cheapest stove) for joining the study. All households in Uganda and Vietnam accepted to purchase one of the provided improved stoves. In Kyrgyzstan, the cookstoves/heaters were donated by the World Bank in exchange for participating in the data collection. Two research assistants (RAs) (members of the local FRESH AIR team) received an intensive 5-day training about the HAP monitoring. The assessment of the questionnaires was performed by trained healthcare workers (Uganda), by medical students (Vietnam) or by the FRESH AIR team (Kyrgyzstan). Effectiveness is described in the previous paragraphs.

Implementation was mostly conducted according to the initial plan. After being trained on awareness of the damaging effects of HAP, the households were able to make a choice between three to four cookstoves/heaters they thought to be the most suitable for them (Supplementary Fig. 1). In Uganda, 35% chose the *ILF Rural Wood* stove, 39% the Berkeley–Darfur stove and 26% the *Biolite* stove. The Berkeley–Darfur stove was chosen because it worked well in windy conditions (villages in open areas). In Vietnam, 12.1% chose the *Greengen* stove (model *The Le Xanh*), 5.4% the *Tien Manh* stove, 55.4% the *Solar Serve 3G* stove and 27% the *Green Bamboo* stove (Model *Tre Xanh*). The Solar Serve 3G stove was most popular because of its convenience and easy-to-use for different fuels. In Kyrgyzstan, 50% chose Model 4 and 50% Model 2.5, influenced by the available fuels (Model 5 was not chosen because this model could not be used for cooking).

Maintenance is related to the acceptability and long-term use of the improved cookstoves/heaters by the community members. In all three countries, the participants were pleased with the new cookstoves/heaters and almost everybody recommended the stoves to others (Table 4). The majority of participants reported the stoves produced less smoke, needed less fuel and took less time to heat. In Uganda, starting the fire was not easy, but once it was lit, there were no problems. Striking remarks from users about the stoves in Uganda were “we use less firewood and charcoal, hence reduce the destruction of the environment”. In Vietnam, the charger of one of the two cookstoves using electricity (Tre Xanh stove) had to be replaced in four households. In Uganda and Kyrgyzstan, not one of the cookstoves/heaters broke down or had to be fixed. The participants in Uganda were most satisfied with the Berkeley–Darfur stove (satisfaction score 9.7), followed by the *ILF Rural Wood* stove and the *Biolite Home* stove (satisfaction score 9.3 and 8.9, respectively). The participants in Vietnam were most satisfied with the *Tien Manh* stove (satisfaction score 9.0), followed by the 3G stove, *Tre Xanh* stove and *The He Xanh* stove (satisfaction score 8.7, 8.3 and 6.9, respectively). Nevertheless, the stoves in Vietnam were considered to be too small and users perceived to have a limited choice of fuels. This resulted in ‘stove stacking’, i.e. parallel use of multiple types of fuels, cookstoves and other pollution sources in a single household. This was encountered in Vietnam among 85.5% of households, and to a lesser extent in Uganda (21%) and Kyrgyzstan (15%).^20,21^ In Kyrgyzstan, people were impressed with the high quality of the heaters, particularly Model 4 (satisfaction score 9.1 compared with Model 2.5, which scored 7.8). A business model was made in Uganda and Vietnam in close collaboration with the energy providers to implement the improved cookstoves. The business model described the different tasks involved in the implementation, and the overall resources needed to support the implementation effort. In Kyrgyzstan, a business model was already made by the energy provider, the World Bank. All these aspects are expected to contribute to long-term and sustained use of the new stoves and heaters.

**Table 4:** Opinions about the improved cookstoves/heaters in Uganda, Vietnam and Kyrgyzstan

		Uganda	Vietnam	Kyrgyzstan
N of households (absolute)		90	76	20
Are you satisfied using the new stove/heater? (score 1–10 with 95%CI)		9.3 (9.1–9.6)	8.4 (8.0–8.8)	8.6 (8.0–9.2)
How confident are you using the new stove/heater? (score 1–10 with 95%CI)		9.5 (9.2–9.7)	9.0 (8.8–9.3)	8.6 (8.0–9.2)
How important is it to use the new stove/heater? (score 1–10 with 95%CI)		9.6 (9.4–9.8)	8.0 (7.6–8.5)	8.6 (8.0–9.2)
Do you recommend the new stove/heater?		98.9	89.8	100
Reasons:	less time cooking	90.0	63.7	100
	less fuel needed	90.0	72.5	100
	tastes better	49.0	16.3	55.5
	less smoke	83.0	67.5	81.0
	easier to clean	59.0	58.8	75.0
	better for health	76.0	46.3	90.0
	not expensive	61.0	13.8	85.0

All data are percentages unless stated otherwise; score 1–10 means 1 = very bad or none at all and 10 = excellent or extremely good

*CI* confidence interval

## Discussion

### Main findings

Before the implementation of locally tailored cookstoves/heaters, people in rural Uganda, Vietnam and Kyrgyzstan had considerable amounts of *daily respiratory symptoms* and *symptoms during cooking*. After implementation, many respiratory symptoms diminished significantly in Uganda and Kyrgyzstan, and to a smaller extent in Vietnam. PM_2.5_ exposure decreased after the intervention (significantly in Vietnam and Kyrgyzstan, and borderline significantly in Uganda), but still remained above the WHO air quality guidelines.^15^ Particularly in Uganda, the background and the maximum PM_2.5_ levels still remained high. Mean CO-levels were, and remained, relatively low.^15^ In all three countries, the communities showed a high acceptance of the new cookstoves/heaters.

### Interpretation and comparison with previous studies

This study shows that implementing improved cookstoves/heaters was associated with lower exposures and improved short-term health benefits. Yet, short-term health benefits in Uganda and Kyrgyzstan were higher than in Vietnam. In Vietnam, the high population density in the rural areas could have exposed the villagers to additional ambient air pollution, causing more symptoms. Although ‘stove stacking’ occurred frequently in Vietnam, their mean PM_2.5_ level was the lowest. There seems to be a difference between the intervention of cookstoves and heaters (which were used for cooking as well): all the heaters in Kyrgyzstan had a chimney and this was not the case with the cookstoves. This could be a reason why in Kyrgyzstan *symptoms during cooking* almost completely disappeared and the PM_2.5_-values substantially decreased to just above the WHO air quality guidelines. In Vietnam, the association between improved cookstoves and health outcomes was less strong. This could possibly be explained by our observation that in Vietnam many men continued to smoke tobacco inside their homes, thereby increasing pollution concentrations, in contrast to Uganda and Kyrgyzstan. Furthermore, mosquito coils were frequently used in Vietnam (74.7% of households), which was not the case in Uganda (6%) and Kyrgyzstan (0%).^22,23^ Absolute PM_2.5_-values were highest in Uganda and we speculate that this could be explained by the high cooking time and frequencies (see Supplementary Table 1) and a relatively high background pollution caused by dust, burning of rubbish, cooking fires from the neighbourhood, fumes from kerosene-based lamps, running diesel generators and grain mills amongst others.^24^ Of note, we observed a decrease in missed school days among children in Uganda and Kyrgyzstan, but an increase in Vietnam. Yet, given the relatively low absolute absence and small numbers per country, single cases with high absence could drive these results and therefore careful interpretation is warranted.

Previous reviews about the effect of interventions to reduce HAP exposure showed a great variety in results of which some driven by cultural and geographical differences.^11,12^ In line with our results, reductions of PM and CO were observed (but almost all still exceeding the WHO air quality guidelines), as well as a decrease of respiratory symptoms. Data effects on other health outcomes such as lung function and incidence of asthma, COPD and lung cancer were inconclusive.^10–12^ First, this limited success of intervention programmes may have been provoked by a lack of implementation or maintenance of the intervention, a too short follow-up time or insufficient HAP risk awareness within the communities.^4^ Second, one could wonder whether health effects are only related to mean or maximum PM_2.5_ or CO-levels. Pollutants with the strongest evidence for public health concern include, aside from PM_2.5_ and CO, black carbon, nitrogen oxides (NO_x_), sulphur oxides (SO_x_), volatile organic compounds and ozone, https://www.who.int/airpollution/ambient/pollutants/en/, https://www.who.int/airpollution/household/pollutants/en/, https://www.who.int/airpollution/ambient/health-impacts/en/. As such, differences in achieved health effects can occur as a result of differences in short- and long-term exposures to combinations of various pollutants that are not always measured concomitantly and consistently across studies.^8^ Third, HAP exposure impacts lung development already in utero, during childhood and early adulthood, resulting in a lower maximum attained lung function later in life. Therefore, lung health benefits from improved cookstoves/heaters should perhaps be measured in the next generation, which can benefit from lower exposures during these crucial phases during their lung development.^25^ Lastly, BOLD results remind us to remain alert for other causes of airflow obstruction than HAP.^26–28^

### Strengths and limitations

A strength of this study was that we conducted the project in three totally different countries, each with their own political and health care infrastructures, landscapes, weather conditions, cultures and traditions, and that we adapted the intervention to local situations and needs. The measurement and outcomes methodology was standardised, while each country had a choice of different cookstoves/heaters, chosen in close collaboration with locally working energy distributors. In Kyrgyzstan, the heaters were even manufactured locally in cooperation with the community, under the guidance of *the World Bank*. Furthermore, the participants were, prior to the intervention, fully aware of the detrimental effects of biomass smoke, taught during the FRESH AIR awareness programme. Some limitations also need to be mentioned. As an implementation study, we had no control group and did not power the study on all measured outcomes. Therefore, we were not able to draw any conclusions on possible causality and confounding factors (such as background pollution) may have driven some of our findings. All the health outcomes, such as *respiratory symptoms*, *symptoms during cooking* and *chest infections*, *CCQ score* and *MRC score* were self-reported and could be susceptible to reporting bias to please the intervention teams. This applied for *type of fuel* and *time needed for cooking*, both indoors and outdoors, as well; these important elements for a successful implementation were subjectively measured in a questionnaire. Besides, not all participants could be followed-up until their second measurement. This could have resulted in selection bias towards the more motivated people. Also, due to economic restrictions and lack of proper pilot data, we could not make formal sample size calculations.

### Recommendations for future studies, practice and policy

There is a need for researchers to reconsider the evaluation in which interventions of improved cookstoves/heaters are designed. Levels of ambient air pollution in the neighbourhood (vehicle exhaust, road dust and local industries) and other confounding sources of exposure to pollutants should be assessed as well.^11,29^ Other non-combustion sources could be moisture build-up, mould and bacterial growth in the houses https://www.who.int/airpollution/household/pollutants/noncombustion/en/.

Even though the postintervention levels of pollution must ideally be below the WHO air quality guidelines, it is important to understand the local perspective, taking into account both socioeconomic and cultural factors.^11,30^ Rural areas in most LMICs lie behind in the transition to cleaner fuels. Still, the short-term effects of reducing HAP exposure, as shown in this study, may encourage communities to change their cooking methods, including the use of clean fuels. Engagement of the community and their leaders in the process of implementation is crucial. Furthermore, HAP is generally linked to poverty.^5,8^ Therefore, other important household health interventions, such as the improvement of poor living conditions, sanitation and nutrition, will demand a new multidisciplinary approach, involving health policymakers and the local government, as well as international partners. Understanding the cultural traditions and the social-economic factors, as well as the health system and the governmental system, are vital for making any intervention successful. Due to extensive FRESH AIR fieldwork exploring local beliefs and perception in their local context, we were aware of barriers and facilitators to the implementation of the cookstoves/heaters. Lastly, as outlined above, health benefits should preferably be measured on a longer term, so that the impact on e.g. lung development can be considered.^25^

In conclusion, the health impact of implementing improved cookstoves/heaters in Uganda, Vietnam and Kyrgyzstan using a tailored approach was considerable. Nevertheless, PM_2.5_ levels remained above the WHO air quality guidelines. The programme was acceptable among the communities. Involving community members and other stakeholders throughout the entire process is a key to make the implementation of improved cookstoves/heaters successful.

## Methods

### Study design

This was an interventional implementation study in three LMICs with a baseline and two postintervention analyses. This was a part of the FRESH AIR (Free Respiratory Evaluation and Smoke exposure reduction by primary Health care Integrated groups) research programme, exploring prevention, diagnosis and treatment of chronic respiratory diseases in resource-poor settings.^31^

### Setting

Uganda, Vietnam and Kyrgyzstan were selected because they represent populations with different geographical and climatic contexts, and exposure to high levels of HAP combined with other risk factors, ^32,33^ http://www.wpro.who.int/vietnam/mediacentre/releases/2018/air_pollution_vietnam/en/. Individual study settings are described below.

#### Uganda

Our study was conducted in the Masindi district, which is located in the mid-west part of Uganda. This area has a predominantly rural population and the majority of residents have been exposed to elevated levels of HAP for their entire lives.^32^

#### Vietnam

The study was implemented in the Can Giuoc district of the Long An province, south of Ho Chi Minh City. Within this rural population, 75% of the households use biomass fuels for cooking.^34^

#### Kyrgyzstan

The Naryn region (2000–3600 metres above sea level) consists of a mostly rural and semi-nomadic population. In most households, biomass fuels are used for cooking and heating.^33^ Because of the long duration of the cold season in these highlands (8–9 months), almost all the families are forced to cook food inside the premises.

### Population and sample size

In each setting, up to ten villages were randomly selected based on the condition they were using a traditional way of cooking. Subsequently, in each village, ten households were randomly chosen. The overall sample size was constrained by budget considerations, namely the cost and level associated with collecting personal pollutant measurements (i.e. particulate matter). Given the focus on implementation, we aimed to include as many participants as our budget allowed, resulting in a convenience sample in each of the three countries (no formal sample size calculation was made).

### Situational analysis

Before the introduction of an intervention, a local situational analysis was performed to understand each setting’s environment and behaviour patterns, particularly their heating, cooking and tobacco-smoking habits. The feasibility and acceptability of different types of cookstoves/heaters and other solutions to reduce exposure to HAP were expected to be highly dependent on the local context, including factors such as climate, availability of different fuels, energy infrastructure, local tradition and cultural practices around cooking, household design and income. All issues were extensively discussed by the local FRESH AIR team in stakeholder group meetings. The number of participants in these meetings varied per country but included energy providers (each had their own meeting with the different cookstoves/heater manufacturers), healthcare workers conducting the survey, the director of the district hospital, the district health officer, the local government, villagers with their village leader and community health worker.

### Description of intervention

First, all villagers were educated about the detrimental effects of tobacco smoking and exposure to HAP by the FRESH AIR Horizon 2020 awareness programme.^31^ Subsequently, after the baseline period, all selected households were provided with improved cookstoves/heaters by energy providers working for many years in the concerned country: *EnDev* (*Energising Development*) in Uganda, *SNV (Netherlands Development Organisation*) in Vietnam and *the World Bank* in Kyrgyzstan. All stoves/heaters were locally manufactured, performance tested and found to be of high quality.

### Description of measurements

To measure the clinical, social and environmental effects of the intervention, a combination of questionnaires and active personal exposure monitoring of pollutants were used. Questionnaires were extensively discussed with local researchers. Their feedback led to further adjustments and eventually a definitive version. All questionnaires were translated to the local language(s) and translated back to English again to double-check the translations. The content of the questionnaires was exactly the same in each country. There was an extensive closed and open-ended household questionnaire (for each household) and a health questionnaire (for the adults and two youngest children), that included validated existing health status tools: the Medical Research Council-breathlessness (MRC) scale and Clinical COPD Questionnaire (CCQ).^35,36^ A qualitative questionnaire was used after intervention with participating households to explore the acceptability of the new cookstoves and heaters.

Personal exposure of particulate matter with an aerodynamic diameter smaller than 2.5 µg (PM_2.5_) was measured with the MicroPEM (RTI International, United States) and Carbon monoxide (CO) was measured with the EL-USB-CO data logger (Lascar Electronics, United Kingdom), https://www.rti.org/impact/micropem-sensor-measuring-exposure-air-pollution, https://www.lascarelectronics.com/easylog-data-logger-el-usb-co/. The personal PM_2.5_ exposures were measured for 48 h and the MicroPEM was worn by the person responsible for domestic cooking, in most cases the woman; this person also wore a collocated CO logger. The CO exposures of the partner (in most cases the husband) were also monitored. Where possible, the CO exposures of the two youngest children were measured simultaneously with the parents. Two RAs in each country received an intensive 3-day training in the setup, deployment and maintenance of the MicroPEM.

The questionnaires and measurements of pollutants were conducted at baseline and twice after implementing a new stove/heater (at 2 and 6 months in Uganda and Vietnam, and 2 and 12 months in Kyrgyzstan). Note that timing of the long-term follow-up measurement in Kyrgyzstan was different from Vietnam and Uganda in order to re-capture the winter season (in Vietnam and Uganda there are no major winter/summer differences in cooking habits). All completed questionnaire data were uploaded in REDCap (a secure web application for building and managing online surveys and databases).

### Outcomes

The outcomes of this study included effectiveness (environmental and health outcomes), and implementation outcomes (acceptability and process evaluation) as specified below.

- Effect on HAP exposure (personal monitoring of PM_2.5_ and CO)
- Effect on health: outcomes before and after intervention:

- Daily respiratory symptoms (cough, phlegm, wheeze, dyspnoea, asked as ‘yes/no’ questions)
- CCQ score (only in adults)
- Symptoms during cooking (respiratory and non-respiratory, asked as ‘yes/no’ questions)
- MRC-breathlessness score (only in adults)
- School days missed (only in children)
- Chest infections

- Acceptability of improved cooking and heating interventions compared with traditional cooking and heating (measured on a scale from 1–10)
- Implementation

To assess implementation, we performed a process evaluation using the dimensions of the RE-AIM framework to describe how the strategy was delivered and implemented, http://www.re-aim.org/.^19^ The dimensions were *Reach* (who actually participated in the intervention), *Effectiveness* (what were the most important benefits, measured by change in key outcomes), *Adoption* (where was the programme applied and who applied it, measured by what settings and staff take up the intervention), *Implementation* (how consistently was the programme delivered and what adaptions to the original plan were made) and *Maintenance* (when did the initiative become operational, and how long will the initiative be sustained over time, measured by longevity of effects and programme sustainability).^20^

### Analysis

Differences in health and HAP exposure outcomes were compared between baseline and (long-term) after the intervention. Differences were assessed with the Wilcoxon Signed Ranks test for paired continuous variables and the McNemar’s test for paired categorical variables. We considered *p*-values between baseline and second post intervention <0.05 as statistically significant. The PM data were analysed in close collaboration with RTI International. The exposure data were lognormally distributed and, therefore, had to be back-calculated to geometric values. All other analyses were performed separately for adults and children using IBM SPSS Statistics version 25.

### Ethics

Participation in this intervention study was voluntary. Each participant signed an informed consent form, or in case of illiteracy, thumb-printed and signed by the village leader. The study was approved by each local research ethical review board: the Mulago Research and Ethics Committee (971;05/24/2016), the Ho Chi Minh City University of Medicine and Pharmacy (188/DHYD-HD;06/27/2016) and the National Center of Cardiology and Internal Medicine in Bishkek Ethics Committee (5;03/03/2016).

### Reporting summary

Further information on research design is available in the Nature Research Reporting Summary linked to this article.

## Supplementary information

reporting summary

Supplementary Material.

## Data Availability

The datasets generated during and/or analysed during the current study are available from the corresponding author on reasonable request.

## Supplementary information

**Supplementary information** accompanies the paper on the *npj Primary Care Respiratory Medicine* website (10.1038/s41533-019-0144-8).

## References

[1] Fullerton DG, Bruce N, Gordon SB (2008). Indoor air pollution from biomass fuel smoke is a major health concern in the developing world. Trans. R. Soc. Trop. Med. Hyg..

[2] Kurmi OP, Lam KB, Ayres JG (2012). Indoor air pollution and the lung in low- and medium-income countries. Eur. Respir. J..

[3] Torres-Duque Carlos A. (2017). Poverty cannot be inhaled and it is not a genetic condition. How can it be associated with chronic airflow obstruction?. European Respiratory Journal.

[4] Amegah AK, Jaakkola JJ (2016). Household air pollution and the sustainable development goals. Bull. World Health Organ..

[5] Landrigan, P. J. et al. The Lancet Commission on pollution and health. *Lancet* **391**, 462–512 (2018). 10.1016/S0140-6736(17)32345-0 29056410

[6] Gordon SB (2014). Respiratory risks from household air pollution in low and middle income countries. Lancet Respir. Med..

[7] Sood, A. et al. ERS/ATS workshop report on respiratory health effects of household air pollution. *Eur*. *Respir*. *J*. **51**, 1700698 (2018). 10.1183/13993003.00698-2017 PMC7418845 29301918

[8] Thurston, G. D. et al. A joint ERS/ATS policy statement: what constitutes an adverse health effect of air pollution? An analytical framework. *Eur*. *Respir*. *J*. **49**, 1600419 (2017). 10.1183/13993003.00419-2016 PMC5751718 28077473

[9] WHO. *Evaluating Energy and Health Interventions: A Catalogue of Methods*, (World Health Organization, Geneva, 2008).

[10] Thomas, E., Wickramasinghe, K., Mendis, S., Roberts, N. & Foster, C. Improved stove interventions to reduce household air pollution in low and middle income countries: a descriptive systematic review. *BMC Public Health* **15**, 650, 015-2024-7 (2015). 10.1186/s12889-015-2024-7 PMC4499941 26169364

[11] Pope D, Bruce N, Dherani M, Jagoe K, Rehfuess E (2017). Real-life effectiveness of ‘improved’ stoves and clean fuels in reducing PM2.5 and CO: systematic review and meta-analysis. Environ. Int..

[12] Quansah R (2017). Effectiveness of interventions to reduce household air pollution and/or improve health in homes using solid fuel in low-and-middle income countries: a systematic review and meta-analysis. Environ. Int..

[13] Rehfuess EA, Puzzolo E, Stanistreet D, Pope D, Bruce NG (2014). Enablers and barriers to large-scale uptake of improved solid fuel stoves: a systematic review. Environ. Health Perspect..

[14] Thakur M (2018). Impact of improved cookstoves on women’s and child health in low and middle income countries: a systematic review and meta-analysis. Thorax.

[15] World Health Organization. *Indoor air quality guidelines: household fuel combustion*. (World Health Organization, Geneva, 2014). 25577935

[16] Rosenthal J (2015). The real challenge for cookstoves and health: more evidence. Ecohealth.

[17] WHO. Prevention and control of noncommunicable diseases: guidelines for primary health care in low resource settings (2012). 23844451

[18] Glasgow RE, Vogt TM, Boles SM (1999). Evaluating the public health impact of health promotion interventions: the RE-AIM framework. Am. J. Public Health.

[19] Glasgow RE, Estabrooks PE (2018). Pragmatic applications of RE-AIM for health care initiatives in community and clinical settings. Prev. Chronic Dis..

[20] World Health Organization. *Burning opportunity: clean household energy for health, sustainable development, and well-being of women and children* (World Health Organization, Geneva, 2016).

[21] Ruiz-Mercado I, Masera O (2015). Patterns of stove use in the context of fuel-device stacking: rationale and implications. Ecohealth.

[22] Liu W (2003). Mosquito coil emissions and health implications. Environ. Health Perspect..

[23] Hogarh, J. N. et al. Environmental health risks and benefits of the use of mosquito coils as malaria prevention and control strategy. *Malar. J.* **17**, 265, 018-2412-4 (2018). 10.1186/s12936-018-2412-4 PMC6048806 30012143

[24] Mortimer K (2017). A cleaner burning biomass-fuelled cookstove intervention to prevent pneumonia in children under 5 years old in rural Malawi (the Cooking and Pneumonia Study): a cluster randomised controlled trial. Lancet.

[25] Brakema, E. A. et al. COPD’s early origins in low- and middle-income countries: what are the implications of a false start?. *npj PCRM* **29**, 6, 019-0117-y (2019) 10.1038/s41533-019-0117-y PMC6401185 30837469

[26] Burney P (2014). Chronic obstructive pulmonary disease mortality and prevalence: the associations with smoking and poverty–a BOLD analysis. Thorax.

[27] Amaral, A. F. S. et al. Airflow obstruction and use of solid fuels for cooking or heating: BOLD results. *Am*. *J*. *Respir*. *Crit*. *Care Med*. **197**, 595–610 (2018). 10.1164/rccm.201701-0205OC PMC6005234 28895752

[28] Siddharthan T (2018). Association between household air pollution exposure and chronic obstructive pulmonary disease outcomes in 13 low- and middle-income country settings. Am. J. Respir. Crit. Care Med..

[29] Martin WJ (2013). Household air pollution in low- and middle-income countries: health risks and research priorities. PLoS Med..

[30] Rosenthal J (2017). Implementation science to accelerate clean cooking for public health. Environ. Health Perspect..

[31] Cragg L, Williams S, Chavannes NH (2016). FRESH AIR: an implementation research project funded through Horizon 2020 exploring the prevention, diagnosis and treatment of chronic respiratory diseases in low-resource settings. NPJ Prim. Care. Respir. Med..

[32] van Gemert F (2015). Prevalence of chronic obstructive pulmonary disease and associated risk factors in Uganda (FRESH AIR Uganda): a prospective cross-sectional observational study. Lancet Glob. Health.

[33] Brakema Evelyn A., Tabyshova Aizhamal, Kasteleyn Marise J., Molendijk Eveline, van der Kleij Rianne M.J.J., van Boven Job F.M., Emilov Berik, Akmatalieva Meerim, Mademilov Maamed, Numans Mattijs E., Williams Sian, Sooronbaev Talant, Chavannes Niels H. (2019). High COPD prevalence at high altitude: does household air pollution play a role?. European Respiratory Journal.

[34] Nguyen Viet N (2015). The prevalence and patient characteristics of chronic obstructive pulmonary disease in non-smokers in Vietnam and Indonesia: An observational survey. Respirology.

[35] Bestall JC (1999). Usefulness of the Medical Research Council (MRC) dyspnoea scale as a measure of disability in patients with chronic obstructive pulmonary disease. Thorax.

[36] van der Molen T (2003). Development, validity and responsiveness of the clinical COPD questionnaire. Health Qual. Life Outcomes.

