# Supplementary material for: Effects and acceptability of implementing improved cookstoves and heaters to reduce household air pollution: a FRESH AIR study
Source: NPJ Prim Care Respir Med. 2019 Aug 15;29:32. doi: 10.1038/s41533-019-0144-8 (PMC6695425; doi:10.1038/s41533-019-0144-8)
Supplement: Supplementary file 2 — Supplementary Material. [file 41533_2019_144_MOESM2_ESM.pdf]

		Uganda	Vietnam	Kyrgyzstan
Number of households (absolute)		100	80	20
biomass for cooking		100	98.8	95.0
biomass for heating		64.0	10.0	50.0
type of stove	open fire	88.0	12.4	0
	single pot stove	7.0	0	5.0
	multiple pot stove	0	8.8	95.0
	surrounded fire	3.0	70.0	0
	griddle stove	2.0	8.8	0
	gas stove	0	0	0
	electric stove	0	0	15
fuel for cooking	wood	89.0	58.8	95.0
	dung	0	1.3	55.0
	grass	53.0	18.8	0
	twigs	25.0	83.8	0
	crop residues	44.0	80.0	0
	charcoal	37.0	7.5	0
	coal	0	2.5	55.0
	kerosene	11.0	5.0	0
	natural gas	0	0	0
	electricity	1.0	0	15
number of meals a day		2.8	2.3	1.1
hours a day cooking indoors		4.0	1.9	1.7
hours a day cooking outdoors		2.5	0.5	0.6
how is smoke removed?	chimney	0	17.5	100
	hood	8.1	1.3	0
	neither	91.9	81.2	0
where is cooking done?	same room as living room	1.0	3.8	45.0
	separate room	9.0	64.6	55.0
	separate building	62.0	24.1	0
	outdoors	3.0	5.0	0
	combination	3.0	0	1.6
ways to reduce smoke	fuel drying	31.0	93.6	0
	use pot lids	8.0	11.6	45.0
	maintenance	17.0	34.6	50.0
	keep children away	64.0	52.6	50.0
tobacco smoking inside		24.0	67.5	20.0

All data are percentages unless stated otherwise;

Supplementary Table 1: Baseline cooking, heating and smoking data before intervention of clean cookstoves/heaters in Uganda, Vietnam and Kyrgyzstan

Uganda

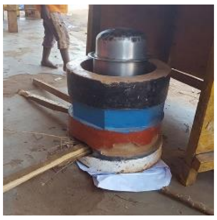

Shielded Fire stove
44% fuel saving
Price: € 2-4

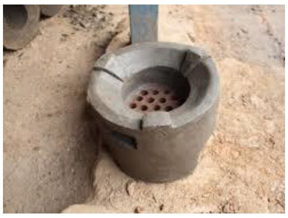

ILF Rural Wood stove
47% fuel saving
Price: € 4

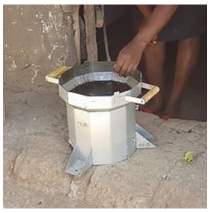

Berkeley Darfur stove
60% fuel saving
Price: € 20

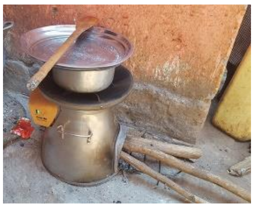

Biolite Home stove
60% fuel saving
Price: € 60

Vietnam

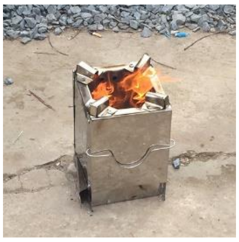

The He Xanh stove
40% fuel saving
Price: € 6

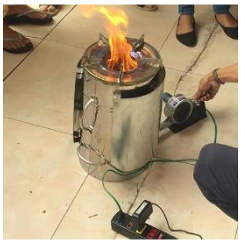

Tien Manh stove
40% fuel saving
Price: € 8.50

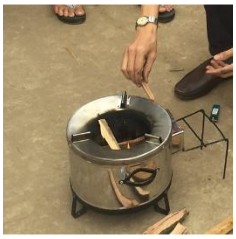

Solar Serve 3G stove
41% fuel saving
Price: € 15

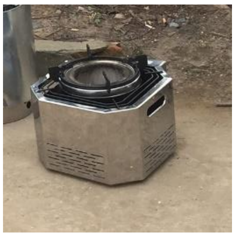

Tre Xanh stove
64% fuel saving
Price: € 17

Kyrgyzstan

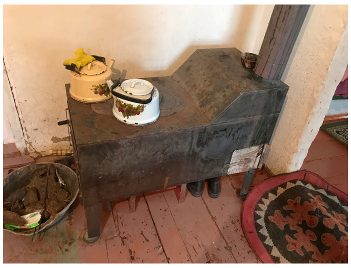

Model 2.5
For heating and cooking
Fuel: coal
Price: € 190

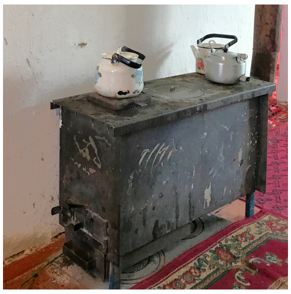

Model 4
For heatimg and cooking
Fuel: coal and dung
Price: € 130

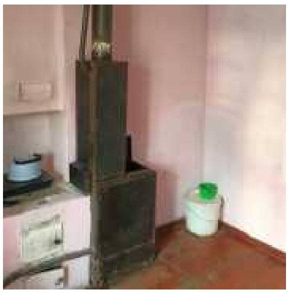

Model 5
Boiler for water heating system
Fuel: coal
Price: € 250

Supplementary Figure 1: The different cookstoves/heaters presented to the participants

Source of images in Supplementary Figure 1

Uganda:
Shielded Fires stove: image taken by the main author
ILF Rural wood stove: image taken by the main author
Berkeley Darfur stove: image taken by the main author
Biolite Home stove: image taken by the main author

Vietnam:
The He Xanh stove: image taken by the FRESH AIR team in Vietnam
Tien Manh stove: image taken by the FRESH AIR team in Vietnam
Solar Serve 3G stove: image taken by the FRESH AIR team in Vietnam
Tre Xanh stove: image taken by the FRESH AIR team in Vietnam

Kyrgyzstan:

Model 2.5: image taken by the FRESH AIR team in Kyrgyzstan

Model 4: image taken by the FRESH AIR team in Kyrgyzstan

Model 5: image taken by the FRESH AIR team in Kyrgyzstan
